# Evolution of the strength characteristics of briquette and raw coal containing fluid

**DOI:** 10.1038/s41598-023-27908-6

**Published:** 2023-01-11

**Authors:** Feiyan Zhang, Chen Niu, Ying Han

**Affiliations:** 1 College of Safety Science and Engineering, Henan Polytechnic University, Jiaozuo, 454003 China; 2 Collaborative Innovation Center of Coal Work Safety and Clean High Efficiency Utilization, Jiaozuo, 454003 China; 3 School of Energy Science and Engineering, Henan Polytechnic University, Jiaozuo, 454003 China; 4 State Key Laboratory Cultivation Base for Gas Geology and Gas Control, Henan Polytechnic University, Jiaozuo, 454003 China

**Keywords:** Solid Earth sciences, Mineralogy

## Abstract

The mechanical properties of coal containing fluid are an important factor affecting the safe mining of soft coal seams. In particular, for class III–V coal, coal and gas outbursts and other dynamic phenomena are prone to occur due to the influences of gas pressure and groundwater, which seriously threaten the safety and lives of field workers. However, briquette samples are usually used in place of raw coal in laboratory tests conducted on class III–V coal samples. Whether the research conclusions for briquette and raw coal are consistent and whether briquette coal can replace raw coal in research on strength characteristics need to be further verified. In this paper, the evolution of the strength characteristics of fluid-bearing briquette coal and raw coal is studied. The strength characteristics, instability failure characteristics, and acoustic emission characteristics of raw coal and briquette coal under uniaxial and triaxial compression are analyzed in detail. In addition, the influence of the water content and pore pressure on the strength characteristics of class III–V raw coal and briquette coal is further studied. The results show that the failure characteristic of raw coal is overall brittle failure, mainly axial splitting failure, whereas that of briquette is overall ductile failure, mainly cone-shaped continuous spalling. The strength parameters of the raw coal and briquette coal improve under confining pressure, but the internal difference in the raw coal is significantly reduced. The cohesion of the raw coal sample initially increases and then decreases with increasing water content, and the internal friction angle increases with increasing water content. In addition, it is verified that the strength, cohesion, elastic modulus, and deformation modulus of the briquette decrease with increasing pore pressure under different pore pressures, but the strength difference of the class III–V coal decreases under increasing pore pressure. Based on the abovementioned results, the strength parameters of a coal body are estimated using the Hoek–Brown (H–B) criterion. Based on a comparison of the strength parameters of the coal sample and coal body, the estimated strength parameters of the coal body are closer to the actual values on site.

## Introduction

The mechanical properties of coal containing fluid are an important factor affecting the safe mining of soft coal seams, especially for class III–V coal. Under the influences of gas pressure, groundwater, and other factors, coal and gas outbursts and other dynamic phenomena are prone to occur, which seriously threaten the safety and lives of field workers. However, briquette samples are usually used in laboratory tests of class III–V coal samples. Whether the research conclusions for briquette and raw coal are consistent and whether a briquette can replace raw coal in research on strength characteristics remain to be verified. For a long time, many researchers in China and abroad have carried out numerous in-depth, detailed studies on the mechanical properties of coal containing fluid. The coal sample is an important part of the coal body. The research objects of the mechanical properties of a coal sample are mainly divided into two types: raw coal and briquettes. For class I and II coal, the structure of the coal body is relatively hard, providing the necessary conditions to drill a raw coal sample, so raw coal samples are usually used in such research. For class III–V coal, the preparation of raw coal samples is difficult, and briquettes are much easier to obtain than raw coal samples, so briquette samples are more commonly used in such research. However, a briquette is a secondary briquetting coal sample, which destroys the structural characteristics of the raw coal sample itself. Class III–V coal is relatively broken, and its characteristics are similar to that of the bulk structure. Therefore, the level of the difference between coal samples of three types and whether a briquette can replace raw coal in research on strength characteristics still need to be verified.

Flow-bearing coal and the raw coal have been extensively studied. For example, Yang Ke et al.^1^ conducted dynamic splitting tests under impact loading on different water-cut coal samples. The energy dissipation characteristics of the coal samples during the failure process were obtained for different water contents, and the influence of the water content on the fractal dimension of the broken coal samples was analyzed. The fractal dimension of the coal samples increased with increasing loading pressure, and the rate of increase slowed. Under the same loading pressure, the fractal dimension of the saturated coal sample was the largest, and that of the dry coal sample was the smallest. Lai Xingping^2^ carried out uniaxial compression tests on coal and rock samples with different water contents and found that under uniaxial compression, the failure mode of the coal and rock samples with different water contents was shear failure, and the shear crack tended to become more complex with increasing water content. Yubing Liu^3^ studied the evolution of the directional permeability of complete coal and fractured coal under different simulated geological conditions. The existence of water in coal can reduce the permeability by an order of magnitude, and in coal samples with rough macrofractures, the permeability decreases significantly. Lu Aihong^4^ discussed the influence of the water content on the mechanical properties and energy dissipation of a rock mass under a dynamic load. As the water content increased from 0 to 2.58%, the crushing rate of the large particles decreased and the small particle crushing rate gradually increased. When the water content was 2.01–2.58%, the fractal dimension increased linearly, indicating that the higher the water content, the larger the fractal dimension of the broken sandstone. The energy calculation results revealed that the energy of the sandstone sample reached the peak value when the water content was 0%. When the water content was 2.01–2.58%, the reflection energy increased, while the transmission energy and dissipation energy decreased linearly. In addition, Qiangling Yao^5^ analyzed the influence of the water content on the strength and deformation characteristics of specimens. As the water content of the coal sample increased, the total stress–strain curve exhibited the characteristics of plastic deformation. There is a positive linear relationship between the peak strain and moisture content and a negative linear relationship between the compressive strength and moisture content. Wang Wen^6^ discussed the mechanical characteristics of water-bearing coal samples under combined dynamic and static loading and carried out three-dimensional combined dynamic and static loading and three-dimensional static loading comparative tests on natural coal samples and coal samples saturated for 7 days using the improved split Hopkinson pressure bar (SHPB) and RMT–150 test system. The results revealed that water saturation had a significant effect on the strength of the coal samples, but the strain rate played a primary controlling role. Under medium to high strain rate conditions, the coupling between the fracture water and fracture results in a larger stiffness, and the dynamic strength of a water-saturated coal sample increases under three-dimensional combined dynamic and static loading. Jiang Jingdong et al.^7^ studied the influence of the water content on the mechanical properties and failure mechanism of rocks. They found that the lower the water content, the smaller the confining pressure, the more severe the damage inside the mudstone, and the weaker the plasticity. For a long time, many researchers have carried out relevant research from the perspective of the mechanical strength of coal samples with different water contents, and abundant research results have been obtained^8–11^. However, little attention has been devoted to whether coal with different water contents can replace raw coal in experimental studies and whether the strength characteristics of coal samples with different water contents can reflect the strength characteristics of the coal body. Therefore, in this study, the strength characteristics of flow-bearing coal and raw coal were studied. Through comparative analysis of the strength characteristics, in-stability, and acoustic emission characteristics of raw coal and briquette samples under uniaxial and triaxial compression, the variations in the strength characteristics of briquette and raw coal were studied under different water contents and pore pressures to reveal the evolution of the strength characteristics of flow-bearing coal and raw coal.

## Materials and methods

### Experimental equipment and sample preparation

**Figure 1:**
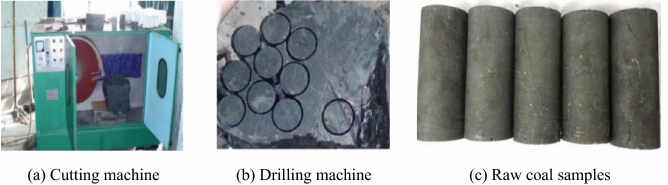
Processing of the raw coal samples.

To compare the strength characteristics of raw coal and briquette samples, considering the difficulty of processing raw coal, a coal sample with complete structure and high hardness that was easy to grind was selected. The coal sample was collected from the No. 3 coal seam in the Huoerxinhe coal mine, Changzhi City, Shanxi Province. A coal sample with a relatively complete structure was obtained, and its failure type was class II coal. First, two parallel planes were cut with a cutting machine to delineate the raw coal sample, and then a core drilling machine was used to drill a cylindrical coal core with a diameter of 50 mm × 100 mm perpendicular to the bedding direction. The two ends of the drilled coal sample were smoothed using a grinder. The sample was allowed to dry naturally in a ventilated location for 48 h, and several coal samples were prepared (Fig. 1).

**Figure 2:**
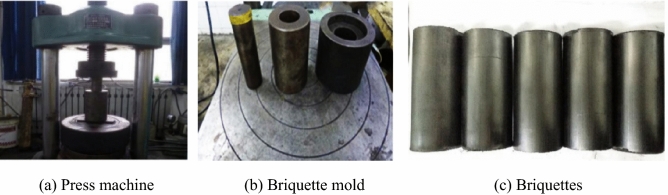
Processing of the briquette samples.

To compare the differences in the strength characteristics of the briquette and raw coal, the coal remaining after drilling the raw coal samples was used to prepare the briquette samples. The raw coal was crushed using a ball mill and unified. Then, it was placed in a standard mold, which was composed of a die cylinder, a pressure piston, and a demolding cylinder. The inner diameter of the die cylinder was 50 mm and the height was 200 mm. The pulverized coal was placed in the mold cylinder, the pressure piston was inserted, a pressure of 100 MPa was applied using the press, the pressure was held for 30 min after it stabilized, and the mold cylinder was placed on the press for demolding. After the briquette sample was obtained, its size was measured and recorded, and it was also allowed to dry naturally in a ventilated location for 48 h. Several briquette samples are shown in Fig. 2.

**Figure 3:**
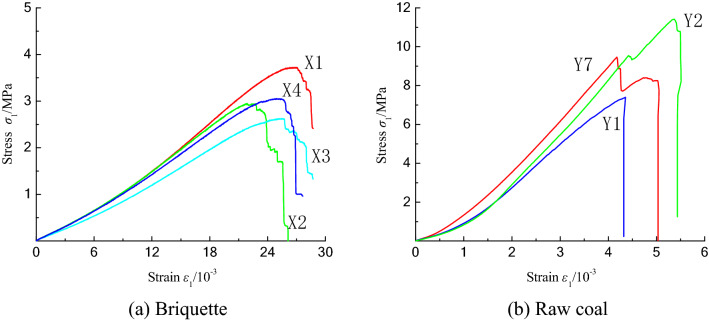
Stress–strain curves of coal samples under uniaxial compression.

The stress–strain curves for the uniaxial compression tests conducted on the two coal samples are shown in Fig. 3. The ordinate is the axial pressure σ_1_, and the abscissa is the strain ε. The change trends of the two curves shown in Fig. 3 are basically the same. Before reaching the peak strength, they undergo a compaction stage, a linear elastic stage, and a plastic stage. The briquette experiences a failure stage, while the raw coal undergoes sudden collapse in this stage.

In the compaction stage, since there is no obvious structural plane in the two types of coal samples, but the internal cracks and pores gradually close under the axial loading, the curves exhibit nonlinear growth, the strain increment decreases with increasing stress, and the curve bends downward. In this stage, the compaction of the raw coal sample is more obvious, and the curvature is larger than that of the briquette sample, which reveals that the closure of the original internal defects in the raw coal sample is more obvious. The curvature of the curve in this stage can reflect the more uniform distribution of the porosity in the briquette sample compared with that of the raw coal. Because of the uniform particle size and high pressure used in the preparation process, the inner part of the briquette sample can be regarded as isotropic. However, the slope of the curve for the raw coal in this stage is caused by the existence of pores, fissures, and cavities, which cause great changes. The raw coal sample enters the linear stage at 14–22% of the peak stress, while the briquette sample enters the elastic stage at 32% of the peak stress. These results reflect the fact that the total number of cracks in the briquette is larger than that in the raw coal, and the compaction stage of the briquette is longer than that of the raw coal.

In the elastic stage, the stress and strain increase linearly. The deformation of the coal sample can be recovered after unloading. However, due to the new microcracks produced in the coal sample under axial loading, it can only be considered to be a quasi-elastic stage. In this stage, the pulverized coal particles in the briquette sample recombine and deform due to the existence of microcracks, and the curve of the briquette sample is more gentle than that of the raw coal. Compared with the raw coal sample, it can be seen that the plasticity of the briquette sample is stronger and the rigidity of the raw coal is stronger. However, this stage reflects the elastic deformation of the coal sample as a whole.

In the plastic stage, which can also be called the unstable crack propagation stage, the stress–strain curve changes from linear to nonlinear growth, the internal cracks begin to expand slightly via friction, the sliding between the particles produces new cracks, and the coal sample exhibits plastic deformation. In this stage, the axial loading curve of the briquette increases.

As the axial load continuously increases, after the raw coal reaches the peak stress, the internal cracks continue to expand in a short time period and finally develop into macrocracks. The ultimate bearing stress of the coal sample decreases rapidly due to the formation of these macrocracks. There is a residual load stress in the briquette sample in this stage, and on the curve, the stress reduction exhibits a zigzag shape, while the curve of the raw coal sample exhibits an abrupt decrease, which is related to the formation of macrocracks in the coal sample.

### Comparative analysis of failure characteristics

**Figure 4:**
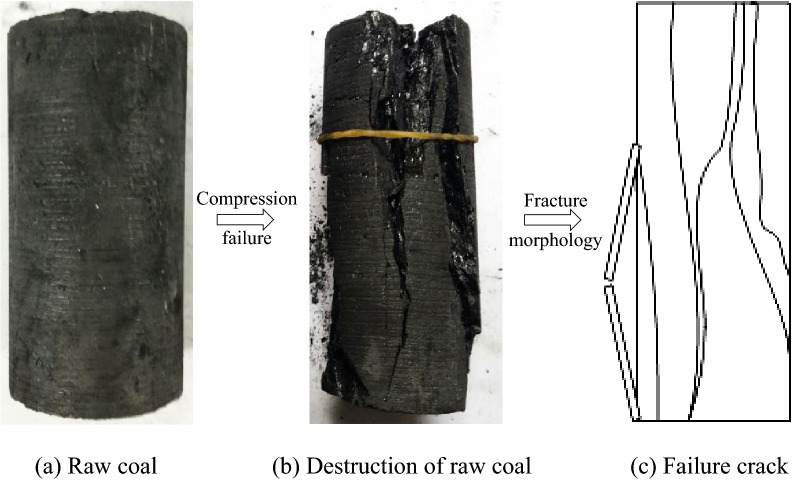
The failure forms of the briquette under uniaxial compression.

Taking a representative raw coal sample as an example, the failure mode of the sample after the uniaxial compression test is shown in Fig. 4. After reaching the peak strength, due to the large stiffness of the raw coal, the stress decreases abruptly, and the stress–strain curve after failure cannot be recorded. This phenomenon is stored in the experimental machine after losing the ultimate bearing stress due to the insufficient stiffness of the experimental machine^12^. When the elastic potential energy of the coal is released suddenly, the coal sample has a large strain.

The small drop in stress near the peak strength^13^ is due to the continuous increase in the tensile stress under the action of shear slip during compression, which makes the sidewall of the coal sample separate and become a compression bar. Under the action of the ultimate tensile stress, the coal sample breaks and collapses, exhibiting brittle failure as a whole. First, the sidewall of the sample breaks, and then the coal sample splits along the through-cutting axial crack.

**Figure 5:**
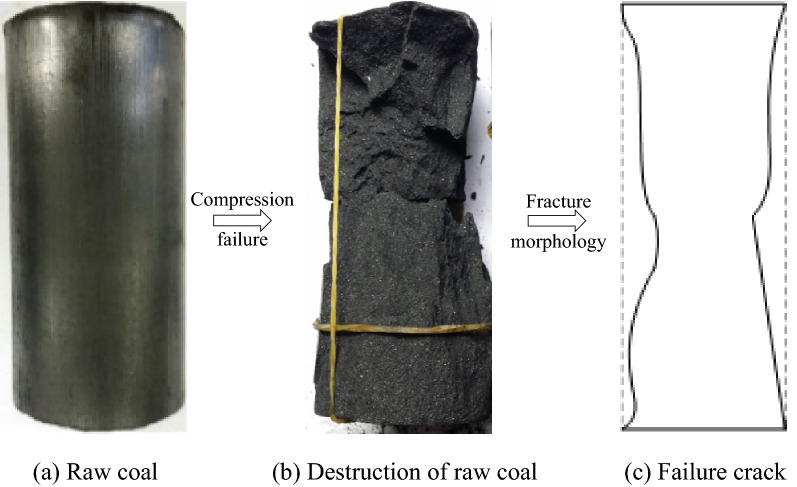
The failure forms of sample X2 under uniaxial compression.

The failure mode of the briquette under uniaxial compression is shown in Fig. 5. The stress–strain curve of the briquette sample in the failure stage exhibits a decreasing saw-toothed pattern. A briquette is formed by mechanical occlusion between the particles and the water-binding effect of the granular coal through pressurization. Although there is a layer of binding water film between the particles due to the presence of water, which can increase the lubrication effect between the particles and is not conducive to briquette formation, a small amount of water is added during the preparation process. After this, the briquette is dried in a ventilated location to make the overall macro-performance of briquette exhibit particle mechanical occlusion. In the loading process without confining pressure, the particles inside the sample are squeezed and rubbed against each other under the load, and some particles break again to form particles of different sizes, which weakens the bite force between the particles. When this force is not sufficient to bear the axial load, the side wall of the sample will continuously spall off from the ends, and the stress will gradually decrease. The stiffness of the cushion block is larger than that of the briquette, and its internal elastic potential energy is smaller and is released with the continuous spalling of the side wall of the coal sample. Due to the end effect, the side wall of the coal sample peels off in a cone shape from the end, and finally the middle part of the coal sample loses its bearing capacity, leading to fracturing.

### Triaxial compression experiment

**Figure 6:**
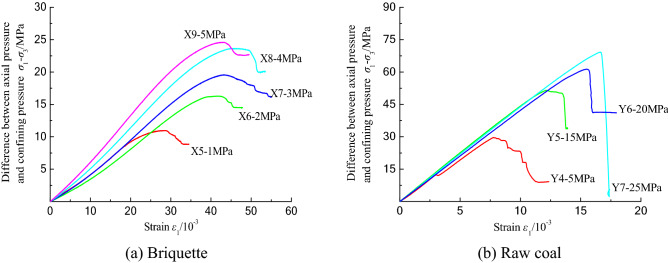
Stress–strain curves of coal samples under uniaxial compression.

The triaxial compression stress–strain curves of the briquette and raw coal samples are shown in Fig. 6. The longitudinal axis in Fig. 6 is the difference between the axial pressure σ_1_ and the confining pressure σ_3_. The change trends of the two groups of curves are roughly the same, and the strength parameters are better than those under uniaxial compression. Under a confining pressure, the compaction stage of the briquette is not obvious, and the cracks and pores in the coal sample are more closely closed than under uniaxial compression. The increase in the friction reduces the shear slip between the particles. Under different confining pressures, the degree of closure of the internal cracks is different. As the confining pressure increases, the peak strength of the coal sample increases, the elastic stage of the raw coal becomes longer, and the plastic stage of the briquette becomes longer. Compared with the results of the uniaxial compression tests, the overall ductility of the raw coal is larger, but the overall failure is still brittle. A briquette is formed by pressing granular coal. The confining pressure increases the plastic deformation of the coal sample, making the particles more densely packed, and the whole sample still undergoes ductile failure. When the confining pressure of the raw coal reaches 15 MPa, the curves of the compaction and linear elastic sections almost coincide. As the confining pressure increases, the elongation of the curves is different, and the internal structure of the coal is dense under the action of the confining pressure. Due to the confining pressure, the coal sample is seriously damaged, and the complete failure mode cannot be obtained.

## Results and discussion

### Influence of moisture content on the strength characteristics of class III–V raw coal

Three sets of typical raw coal samples of classes III, IV, and V with different moisture contents were used for the direct shear tests.

1. The class III, IV, and V coal samples were divided into 12 groups according to the designed moisture contents of 6–28% and were weighed. Based on the moisture contents of the three types of coals, the mass of the water to be added was calculated according to the mass of the coal sample. Water was sprayed on the flat coal sample with a syringe, and then the sample was evenly stirred and sealed in a sample bag for 24 h.
2. After weighing the coal sample for 24 h, Vaseline was applied to the inside of the ring knife and it was weighed. The coal sample was cut using the ring knife, compacted using a rubber hammer and the unified compaction method to control the compactness, and then the surface was repeatedly repaired. The prepared ring knife sample was covered with a plastic cover plate.
3. Twelve samples with the same moisture content were prepared, and three groups of direct shear tests were conducted.

### Analysis of strength characteristics of raw coal with different water contents

#### Effect of moisture content on cohesion of class III–V raw coal

**Figure 7:**
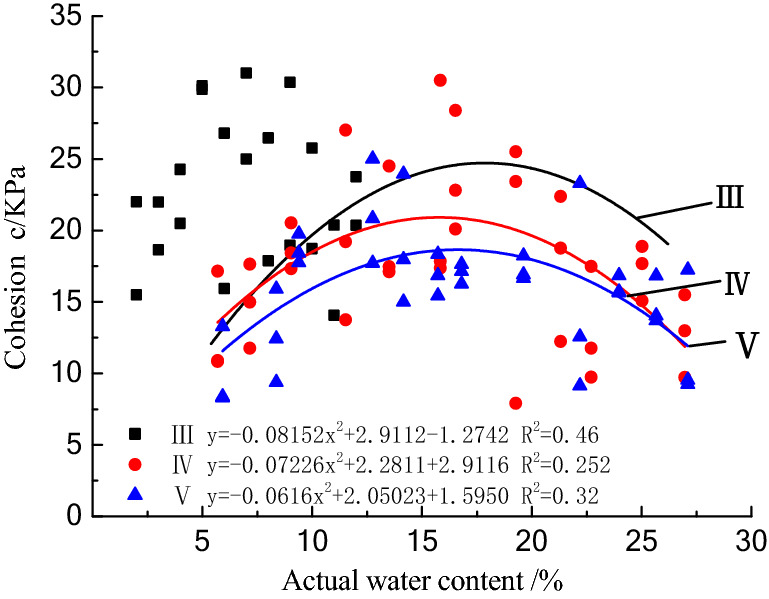
The relationship between the cohesion and moisture content.

The variation in the cohesive force of the class III–V bulk raw coal with water content is shown in Fig. 7. For the three types of coal samples, the cohesive force initially increases and then decreases with increasing water content. The variation range of the cohesive force of the class III coal sample with water content is larger than those of the class IV and V coal samples. The optimal moisture content of class III coal is about 17%, and that of class IV and class V coal is about 15%. The cohesion of class III coal is more sensitive to the moisture content.

The water in the bulk raw coal is mainly free water and bound water. The bound water is tightly adsorbed around the coal particles due to the electrostatic attraction of the particle surfaces, so it cannot flow freely or transfer the hydrostatic pressure^14^. Bound water can be divided into two types according to the distance between the particles: strongly bound water and weakly bound water. Strongly bound water is strongly affected by the electrostatic force, while weakly bound water is less affected and can move freely, so it is an important part of the bound water film. When the moisture content is low, the binding water film is thin, the bonding effect is weak, and an agglomerate structure cannot be formed, so the cohesion is low^15^. Taking class III coal as an example, when the moisture content reaches 0–17%, the water film bonding effect gradually increases, and surface tension forms on the surface of the water film under the capillary effect, which is beneficial for the internal particle bonding of the coal sample. When the moisture content is greater than 17%, the water film between the particles gradually thickens, and the free water also weakens the degree of connection. In addition, due to the increase in the water saturation, the matrix suction of the coal sample gradually decreases^16^, and the cohesion gradually decreases, which also improves the strength of the coal sample.

#### Influence of moisture content on internal friction angle of class III–V raw coal

**Figure 8:**
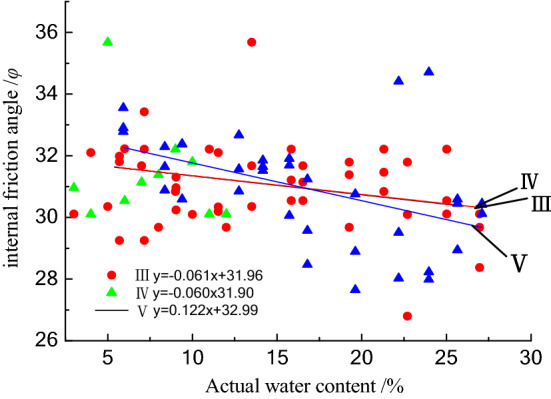
The relationship between the internal friction angle and moisture content.

The relationship between internal the friction angle and water content for the class III–V loose coal samples is shown in Fig. 8. The internal friction angle decreases with increasing water content, but the relationship has a low degree of fitting and no obvious overall correlation. The results show that the internal friction angle of class III and IV coal almost coincides with the fitting curve of the water content, and the internal friction angle of class V coal changes greatly with water content.

The internal friction angle is mainly the embodiment of the friction performance of the coal sample, which mainly overcomes the friction caused by the rough surfaces between the particles. As the water content increases, the bound water film between the particles thickens, weakening the coupling effect between the particles and increasing the lubrication effect between the particles. Thus, the internal friction angle decreases with increasing water content.

### Analysis of strength characteristics of class III–V raw coal with different moisture contents

In the actual coal mining process, water is injected into the soft coal wall to improve the stability of the coal wall and prevent coal and gas outburst accidents. To determine the optimal water content of the coal wall, briquette samples with different water contents were prepared. Five types of briquette samples with water contents of 2%, 4%, 6%, 8%, and 10% were prepared. Because of the small dispersion of the briquette samples, a briquette sample needed to be prepared for each moisture content.

**Figure 9:**
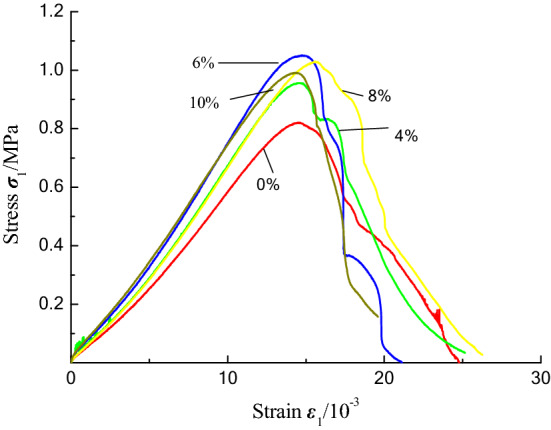
Stress–strain curves of the coal briquette samples with different moisture contents under uniaxial compression.

The uniaxial compression test results for the coal samples with different moisture contents are presented in Table 1, and the uniaxial compression stress–strain curve is shown in Fig. 9.

**Table 1:** The uniaxial compression test results for the coal briquette samples with different moisture contents.

Moisture content (%)	MPa	GPa	GPa
0	0.821	0.066	0.055
2	0.839	0.070	0.059
4	0.956	0.077	0.063
6	1.051	0.084	0.070
8	1.029	0.078	0.063
10	0.991	0.079	0.071

**Figure 10:**
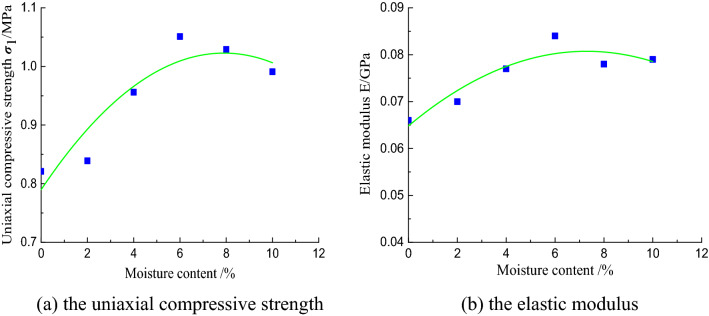
The fitting curves of the moisture content versus (**a**) the uniaxial compressive strength and (**b**) the elastic modulus.

The curve of the uniaxial compressive strength and elastic modulus of the coal samples with different water contents is shown in Fig. 10. The strength and elastic modulus of the briquette increase with increasing water content within a certain range. When the water content is 6%, the strength and elastic modulus of the coal sample reach the maximum values. As the water content increases from 0 to 6%, the strength increases by 28%, and the elastic modulus increases by 27%. When the moisture content is greater than 6%, the strength and elastic modulus of the coal sample exhibit decreasing trends. However, moisture is an essential component, and reasonable water content can increase the cohesiveness between the coal particles during the process of briquette preparation.

The strength of the coal sample is mainly composed of its own cohesive force and the friction force generated by the load^17^. When the water content of the coal sample is low, there is little free water between the particles in the coal sample, and the liquid bridge formed by the combined water almost does not produce an effective force. Under a load, the particles in the coal sample cannot move relative to each other and form a dense structure, which reduces the strength of the sample. At this time, the strength mainly depends on the friction. When the moisture content is too high, the water film between the internal cracks and the particles of the coal sample is thick, which weakens the connection between the particles and decreases the strength of the sample^18^. A reasonable amount of moisture plays a lubricating role among the particles in the coal sample. A briquette is formed under high pressure, which makes it easy for the particles to rub against each other under a load. Small particles enter the spaces between the large particles, and the cohesion generated by the mechanical occlusion between the particles causes the structure of the coal sample to become denser, which causes the strength of coal sample to reach the maximum^19,20^. Therefore, the determination of reasonable moisture content is helpful for improving the strength of coal samples.

## Strength characteristics of briquettes under different pore pressures

To obtain the triaxial strength characteristics of class III–V coal samples under different pore pressures and confining pressures, pore pressures of 0.2 MPa, 0.4 MPa, 0.6 MPa, and 0.8 MPa were selected. To compare the results to the previously presented strength characteristics of a coal sample without pore pressure, pressures of 0.6 MPa, 0.8 MPa, and 1.0 MPa were selected. Finally, the differences in the strength characteristics of class III–V coal samples under the same pore pressure and confining pressure were compared.

The testing system was mainly composed of a RMT-150B rock mechanics testing machine, an improved sample chamber, a high-pressure CO_2_ gas source (for safety reasons, CO_2_ was used instead of CH_4_), and a CY-60 gas pressure sensor.

To allow comparison with the strength characteristics of the coal samples, the same confining pressure and loading method were adopted. Xu Jiang et al.^21^ determined that gas adsorption saturation had little effect on the deformation degree and peak strength of coal samples by conducting comparative experiments under gas and nitrogen conditions. Therefore, whether the coal sample is saturated has little influence on the experimental results. After the coal sample was installed, the confining pressure was applied to the target value, and CO_2_ gas was introduced. After the gas was discharged to the outside of the apparatus through the coal sample and stabilized, the exhaust valve was closed and the experiment was carried out.

**Figure 11:**
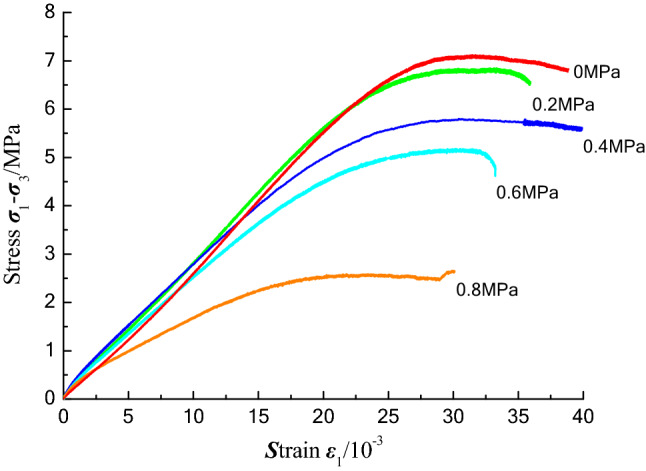
Stress–strain curves of coal specimens with different pore pressures under triaxial compression.

**Figure 12:**
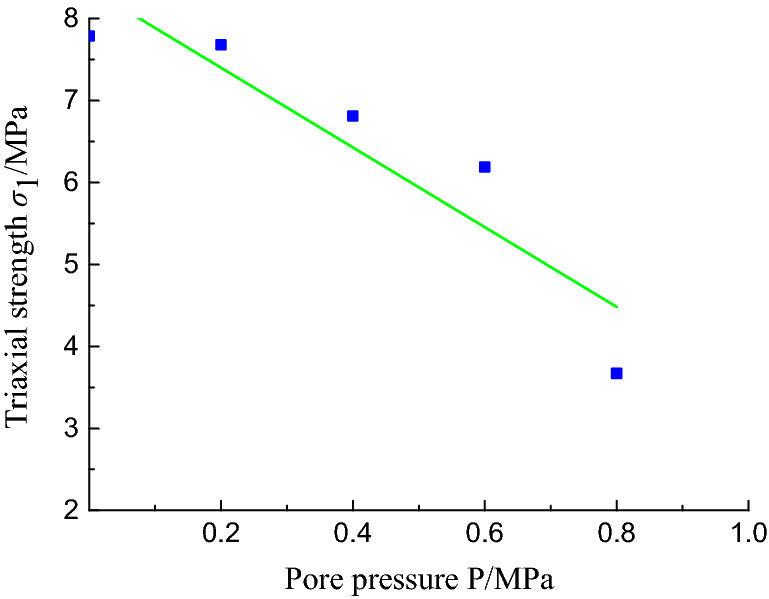
The fitting curve of the triaxial strength versus the pore pressure.

According to the designed experimental scheme, a total of 10 groups of triaxial compression experiments with different confining pressures, gas pressures, and coal sample types were carried out. The experimental results are presented in Table 2. The triaxial stress–strain curve for a constant confining pressure of 1 MPa is shown in Fig. 11, and the fitting curve of the pore pressure versus the strength is shown in Fig. 12.

**Table 2:** Experimental results of triaxial compression tests on coal briquette specimens under different pore pressures.

Coal sample	P (MPa)	σ_3_ (MPa)	σ_1_ (MPa)	E (GPa)	E′ (GPa)
IV	0	1	7.79	0.340	0.350
IV	0.2		7.68	0.290	0.280
IV	0.4		6.81	0.250	0.280
IV	0.6		6.19	0.230	0.250
IV	0.8		3.67	0.140	0.180
IV	0.2	0.8	6.62	0.250	0.250
IV	0.4		6.26	0.240	0.260
IV	0.2	0.6	5.02	0.200	0.230
IV	0.4		4.39	0.170	0.200
III	0.4	1	6.60	0.230	0.230
V	0.4		6.96	0.280	0.280

The strength of the coal sample decreases with increasing pore pressure. As can be seen from Fig. 12, as the pore pressure increases, the duration of the plastic deformation stage of the coal sample increases, which is due to the reinforcement of the fixed confining pressure during the ventilation of the coal sample in the initial stage of the experiment.

The triaxial stress–strain curves for the type IV coal samples under 0.8 MPa and 0 MPa are shown in Table 2. Under the pore pressure, in the compaction stage, the curve of the coal sample bends upward because there is pore pressure in the internal pores in the coal sample in the section OA. At this time, as the axial load increases, the direction of the pore pressure is opposite to that of the confining pressure, and the pore pressure is beneficial to the formation and development of pores, offsetting part of the confining pressure. Therefore, the compaction stage hardly exists. It can be seen from Fig. 11 that as the pore pressure decreases, in the compaction stage, the curve gradually changes from upward bending to downward bending.

**Figure 13:**
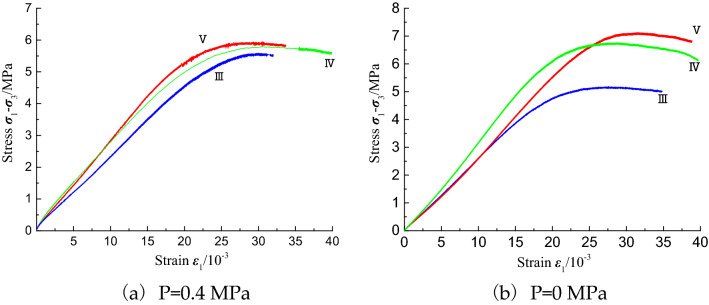
Stress–strain curves of class III–V coal samples under different pore pressures and triaxial compression.

The triaxial compression stress–strain curves for the type III–V coal samples under pore pressure and conventional conditions are shown in Fig. 13. The three coal samples exhibit obvious strength differences in the conventional triaxial compression experiments; however, under pore pressure, the three curves are consistent, and the peak strength and its differences are further reduced. Moreover, under pore pressure, the difference in the internal structures of the three kinds of coal samples is reduced.

## Comparison of strength characteristics of coal samples and coal bodies based on H–B criterion

There is no effective theoretical method to prove the difference in the mechanical environments of briquette coal and raw coal. In a rock mass in the field, the generalized Hoek–Brown (H–B) criterion and geological strength index (GSI) are used to estimate the mechanical parameters of the rock mass and then to study the mechanical properties of the rock mass. Brown conducted a large number of triaxial tests on coal samples and found that the peak strength of coal samples can be estimated using the H–B criterion. As a special rock, the mechanical parameters of coal are measured using rock mechanics experiments. Then the strength parameters of the coal mass are estimated using the generalized H–B criterion to provide a reference for studying the strength characteristics of coal.

Generalized H–B criterion:

$\sigma_{1} = \sigma_{3} + \sigma_{ci} (m_{b} \frac{{\sigma_{3} }}{{\sigma_{ci} }} + s)^{a}$, *σ*_1_ is the maximum principal stress in the case of rock mass failure (MPa); *σ*_3_ is the minimum principal stress in the case of rock mass failure (MPa); *σ*_*ci*_ is the uniaxial compressive strength of the intact rock (MPa); m_b_ is the depreciation of the empirical parameter mi of the complete rock; *a* is the characteristic constant of a jointed rock mass; and *s* reflects the degree of rock fragmentation.

The above-presented uniaxial and triaxial compression data for the raw coal and briquette samples were used to estimate the strength parameters of the coal body using the generalized H–B criteria. The GSI was estimated to be 55 by describing the characteristics of the coal body from which the coal samples were obtained. The strength parameters of the coal body were calculated using the generalized H–B criterion. The data are presented in Table 3.

**Table 3:** Mechanical parameters of coal mass.

Coal	mb	s	a	σ_cm_ (MPa)	σ_3n_ (MPa)	C (MPa)	φ (°)	Em (MPa)
Raw coal	1.54	0.0067	0.500	1.670	0.51	0.74	24.20	6.6
Briquette coal	5.29	0.0067	0.500	1.466	1.53	0.75	25.22	3.8

The main component of the coal body is the coal block. In addition, it includes a structural plane composed of joints and fissures, which divides the coal body into discontinuous coal blocks. The main difference between the raw coal samples and the coal body is that the structural plane increases the complexity of the coal body’s stress and stress deformation, which makes the mechanical characteristics of the two different.

The mechanical parameters of the coal sample and coal body are shown in Table 3. It can be seen from Table 3 that the cohesion, internal friction angle, and uniaxial compressive strength of the raw coal and briquette samples are all greater than the corresponding estimated values for the coal body. Due to the existence of a weak structural plane inside the coal body, the uniaxial compressive strength of the coal body is far lower than that of the coal sample. The uniaxial compressive strength of the coal body estimated using coal samples is roughly three times those of the raw coal samples and briquette samples. There is little difference between the estimated values of the cohesive force and internal friction angle. It can be seen from the formula that the cohesive force is related to the size of the internal friction angle, the strength of the coal sample, and the selection of the GSI value of the coal structure, but the GSI value of the coal structure has a great influence on the estimated value. The estimated value is 55, so the difference between the two is not great. The mechanical parameters of other coal bodies are greatly related to the strength of the coal samples, so the strength parameters estimated using raw coal and briquette samples are different.

Based on the experimental data obtained for the class III–V coal samples, it is estimated that the GSI values of the coal body structure from which the class III–V coal samples were collected are 20, 15, and 5, respectively according to Table 4. The estimated results are shown in Table 4.

**Table 4:** Mechanical parameters of class III–V coal mass.

Coal	m_b_	s	a	σ_cm_ (MPa)	σ_3n_ (MPa)	C (MPa)	φ (°)	Em (MPa)
III	3.90	0	0.55	0.31	6.84	0.32	15.71	0.77
IV	2.33	0	0.58	0.13	4.56	0.28	15.45	0.71
V	1.55	0	0.63	0.10	3.87	0.22	14.94	0.43

**Figure 14:**
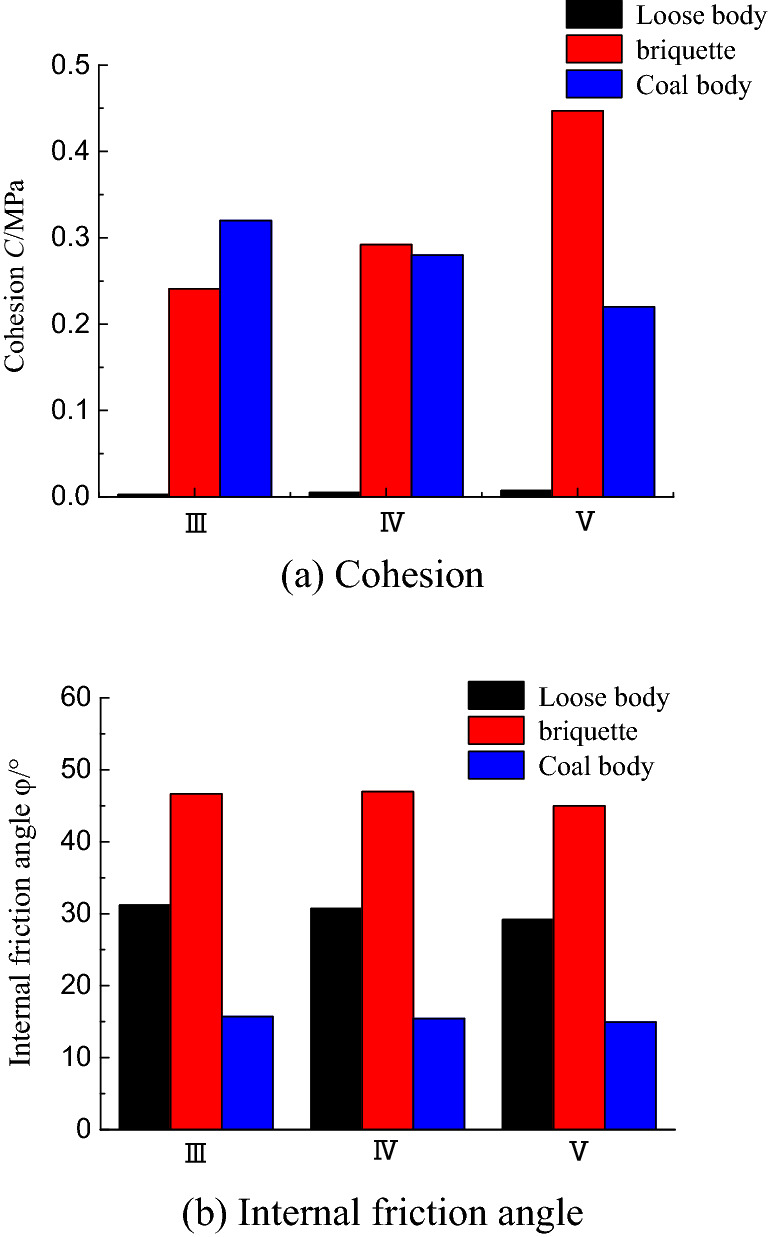
Bar graphs comparing the cohesion and internal friction angles of class III–V coal samples and coal mass.

Table 4 presents the estimated mechanical parameters of the coal samples obtained in this study and the shear strength parameter c of the coal and the coal samples under two conditions. The φ values are compared in Fig. 14. The parameter values of the briquette and bulk coal samples differ greatly from the estimated values. The measured cohesion of the bulk coal samples is too small, and the internal friction angle is too large. The cohesion of the coal samples decreases from class III to class V, while the internal friction angle does not fluctuate significantly. By contrast, the estimated cohesion of the coal samples increases from class III to V, and the internal friction angle fluctuates slightly. The estimated values of the mechanical parameters should be closer to reality and increase the safety and reliability of their use in numerical simulations and engineering applications.

## Conclusions

1. Through uniaxial and triaxial compression tests, the differences between the strength characteristics of raw coal and briquette coal were analyzed. The failure characteristics of raw coal are mainly axial splitting failure and overall brittle failure, while that of briquette coal is mainly ductile failure due to continuous spalling of the side wall in a cone shape. The strength parameters of raw coal and briquette coal improve under confining pressure, and the internal difference in the raw coal is significantly reduced.
2. The cohesion c of the loose coal sample has no obvious correlation with its fractal dimension D, and the internal friction angle φ decreases as a negative exponential function with increasing fractal dimension D. The cohesion of the coal sample initially increases and then decreases with increasing water content, and the internal friction angle decreases with increasing water content. The cohesion of class III coal is more sensitive to changes in the water content, and the internal friction angle of class V coal is the most sensitive to changes in the water content.
3. The strength, elastic modulus, and deformation modulus of the briquette coal initially increase and then decrease with increasing water content.
4. Under different pore pressures, the strength, cohesion, elastic modulus, and deformation modulus of the briquette coal decrease with increasing pore pressure, but under the effect of pore pressure, the strength difference of the class III–V coal samples decreases.
5. Using the H–B criterion, it was found that the strength parameters of the coal samples and the GSI value of the coal structure have a great influence on the accuracy of the estimated values of the coal strength parameters. By comparing the strength parameters of the coal samples and coal bodies, it was found that the estimated values of the coal strength parameters should be closer to the actual situation on site.

## Data Availability

The data that support the findings of this study are available from the corresponding author upon reasonable request.
